# Implementing Health Apps for Digital Public Health – An Implementation Science Approach Adopting the Consolidated Framework for Implementation Research

**DOI:** 10.3389/fpubh.2021.610237

**Published:** 2021-05-07

**Authors:** Julian Wienert, Hajo Zeeb

**Affiliations:** ^1^IU International University of Applied Science, Bad Reichenhall, Germany; ^2^Leibniz Institute for Prevention Research and Epidemiology – BIPS, Bremen, Germany; ^3^Leibniz Science Campus Digital Public Health Bremen, Bremen, Germany; ^4^Department Human and Health Sciences, University of Bremen, Bremen, Germany

**Keywords:** digital public health, digital public health intervention, implementation science, CFIR framework, complex digital health interventions

## Abstract

Apps are becoming an increasingly important component of modern Public Health and health care. However, successful implementation of apps does not come without challenges. The Consolidated Framework for Implementation Research (CFIR) provides a central typology to support the development of implementation theories and the examination of what works where and why in different contexts. The framework offers a reasonable structure for managing complex, interacting, multi-level, and transient states of constructs in the real world: It draws on constructs from other implementation theories and might be used to conduct formative evaluations or build a common body of knowledge for implementation thru various studies and settings. In a synthesis of the original English language text describing the CFIR, an attempt was made to break the constructs down into the shortest possible concise descriptions for the implementation of health care apps in a structured, selective process. The listed key constructs should help to develop successful implementation plans and models for health apps and show the complexity of a successful implementation. As a perspective article, the aim of the current piece is to present a viewpoint on using the CFIR as a potential support for implementing health apps.

## Introduction

When designed, implemented, and used properly, health information technology (HIT) can be a positive enabler to transform the way digital public health is delivered and realized. In fact, HIT interventions for digital public health, for example health apps, provide the chance to increase the performance of health care services, increase their quality, save costs, and successfully involve patients as partners managing their own health care (1). However, if designed and applied inappropriately, such technologies can either be entirely without benefit to the user or add an additional layer of complexity, which can lead to unintended adverse consequences (2). Digital public health interventions (DPHI) render a focus not only on prevention, health protection and promotion. Therefore, DPHI might be defined as a discrete mode of operation for digital technology that is applied to follow and accomplish horizontal and vertical essential public health functions. Hence, DPHI can be implemented within digital applications for health or even whole systems for ICT, also including communication channels such as text messaging systems (3, 4).

Still, insufficient attention has been paid to the implementation and monitoring of health apps that have proven effective in controlled studies on regular health care delivery. One example is apps for depression therapy: Recent meta-analyses confirm the effectiveness of apps that address depression in terms of reduced symptoms, however, little is known about if and how such effective apps are used for prevention or how these apps are implemented into health systems (5, 6). This is important because there is a risk that a program will not be implemented as intended (i.e., implementation failure), which can lead to unsuccessful attempts to achieve the intended intervention effects or even negative intervention effects (e.g., due to lack of uptake) (7, 8). Other risks include frustration and demoralization of health care workers and loss of time in health care delivery that affects team performance in delivering care. This may be the case, for example, when users are interested in integrating apps and digital data into their regular health care, thereby, increasing the workload of health professionals (9–11).

**Figure 1:**
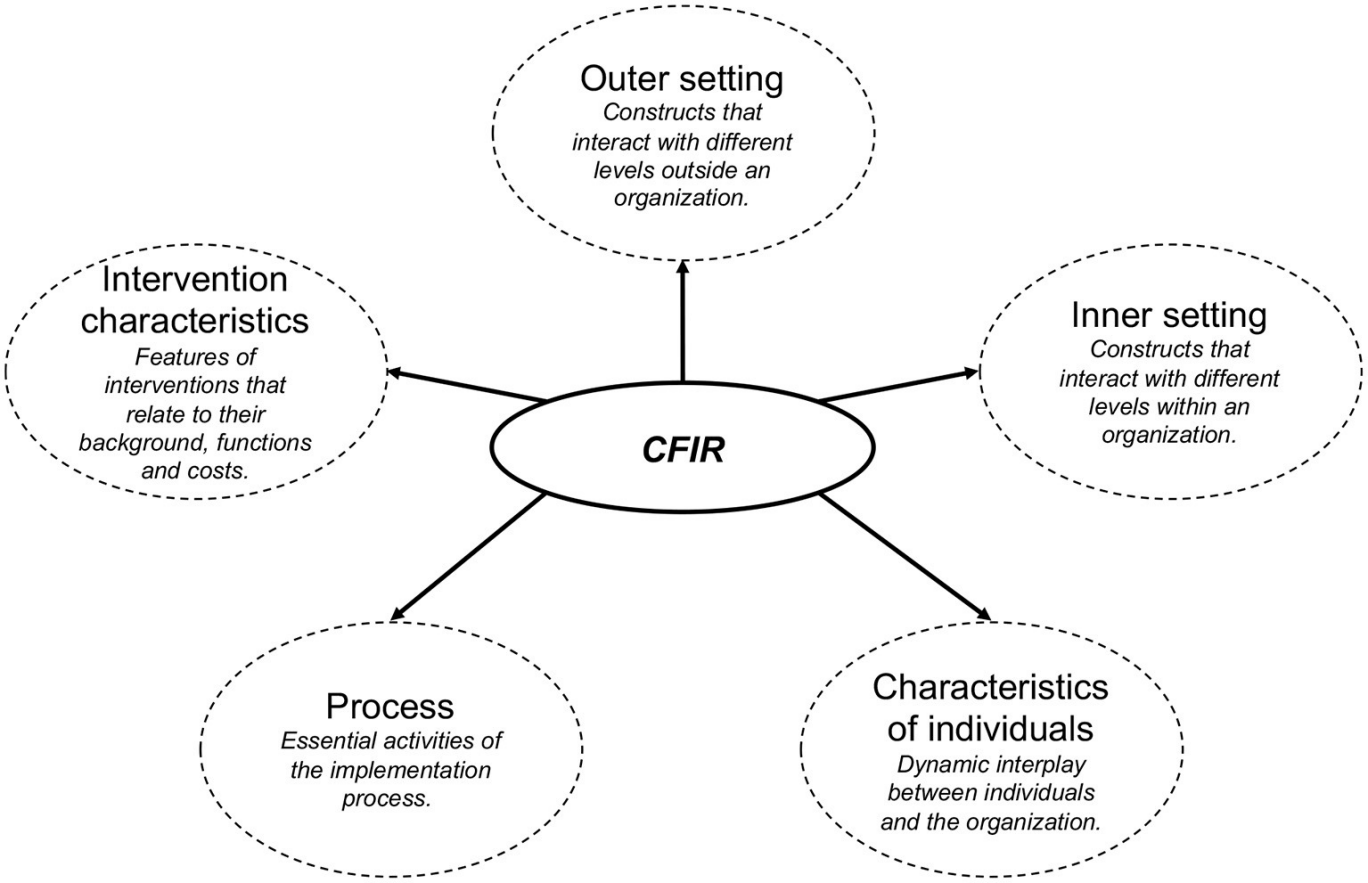
Five domains of the original CFIR with a short description for each domain.

To ensure quality and increase effectiveness of health apps, implementation science provides the necessary repository of ideas and instruments to facilitate implementation, and monitoring of apps. Following Eccles and Mittman (12) implementation science is defined as “the scientific study of methods to promote the systematic uptake of research findings and other evidence-based practices into routine practice, and, hence, to improve the quality and effectiveness of health services.” Among the established instruments, the Consolidated Framework for Implementation Research (CFIR; https://cfirguide.org) (13) provides an overarching typology to support the development of implementation theories and the examination of what works where and why in different contexts. It draws on constructs from other implementation theories and might be used to conduct formative evaluations or build a common body of knowledge for implementation. The framework represents the synthesis of 19 theories associated with implementation science to summarize potential barriers and facilitators of implementation, to ensure consistent use of constructs across studies and to support their comparability. These constructs are broadly subsumed under five domains: (i) Intervention characteristics; (ii) Outer setting; (iii) Inner setting; (iv) Characteristics of individuals; (v) Process (Figure 1). The framework illustrates 39 constructs which reflect the evidence base of intervention features that are most likely to influence their implementation (14).

Understanding which constructs or groups of constructs promote or inhibit adoption, implementation, and maintenance can inform development when planning tailored and testable implementation strategies (15) to balance internal and external validity (16) and push and pull (17). In other words, examining the presence or absence of CFIR constructs can help explain “why” the implementation of a health app was (un-) successful (18).

## Adopting the CFIR for Implementation of Health APPS

Given the rapid development and high dynamics of apps in health, we aimed to map the CFIR constructs to the implementation of health apps, using a shortened description of CFIR constructs. In a synthesis of the original English language text, we broke down the 30 CFIR constructs into the shortest possible concise descriptions for the implementation of apps in a structured process. Descriptions were rephrased to suit the implementation of apps in a broader context (e.g., not only apps for health behavior change), and were paraphrased and shortened (Table 1). A complete comparison of the original and adapted constructs is displayed in Supplement 1. The key constructs of the category “Inner Setting” as well as the construct “Individual identification with organization,” might only be relevant for certain cases, as these specifically address organizations and employees of organizations in which interventions are carried out (e.g., a new app is implemented in an organization).

**Table 1:** Overview of adapted CFIR constructs for the implementation of health apps.

**Adapted construct**	**Adapted construct description**
**Features of the app**	
Source of the app	Clear and transparent description of the app, its functions and sources of the source code (e.g., developer). Preferentially, the source code should be freely available. Clear and transparent description of data usage and data protection.
Evidence strength and quality	Description of the current status of previous, scientific findings on the quality, and validity of the app.
Relative advantage	Demonstration of the positive benefit of the stakeholder's use of the app.
Adaptability	Description of the extent to which core components of an app can be adapted, tailored, refined, or newly developed to individual or local needs (e.g., in the event of different legal regulations or low network coverage).
Feasibility	Ability to trial the app under real conditions (e.g., in the course of a pilot study) and to allow a reversal of the implementation when justified (e.g., when the app is no longer needed or superseded by other developments)
Complexity (practical implementation difficulties)	Perceived implementation difficulties which are reflected in duration, scope, disruptivity, centrality, complexity, and number of steps required for implementation. Increased complexity may be accompanied by increased implementation effort (e.g., due to growing need for content coordination).
Design quality and packaging	The app must be able to meet common design requirements (e.g., verified by design tests).
Costs	Costs of the app and costs related to the implementation of this app, including investment, supply, and opportunity costs. Costs should be in relation to the expected benefits, both for the user and the developer.
**Outer setting**	
User needs and resources	Consideration of the needs and resources of the app's target group (e.g., perceived vulnerability, literacy, language, but also storage space, data volume, availability of hardware, network connection).
Cosmopolitanism	Cooperation and exchange with external developers and stakeholders (e.g., professional associations)
Peer pressure	Other providers are already present on the market with apps, while new providers still have to follow suit. The resulting “peer pressure” can make apps more error prone.
External policies and incentives	Legal regulations, recommendations, or guidelines for the use of the app.
Inner Setting^*^	
Structural characteristics	A stable team, which is entrusted with the development of the app, increases the probability of a successful implementation of the app. Centralization of decision-making autonomy can be negatively associated with innovation. Administrative intensity may be positively associated with innovation. Size, age, maturity, and degree of specialization of the developer might influence implementation as well.
Networks and communications	High social capital within a team, i.e., dimensions of shared vision and information sharing, can contribute to effective implementation through a sense of “team spirit” or “community”.
Culture	Norms and values, or a mindset and culture of an app provider.
Implementation climate	The ability to absorb change, the overall receptivity of people to engage with an app, and the extent to which the use of that app is rewarded, reinforced, and demanded within their organization. Six substructures contribute to a positive climate for app adoption: Enthusiasm for change, compatibility, relative priority, organizational incentives, and rewards, goals and feedback, and learning climate. Readiness to implement: concrete and direct indicators of organizational readiness to implement an app, including leadership commitment, availability of resources, and access to information and knowledge.
**Characteristics of individuals**	
Knowledge and convictions about the app	Perception of a credible external presentation of the app, as well as knowledge of the users how to handle it.
Self-efficacy	The more confident persons feel about their ability to use the app effectively, the higher their self-efficacy. People with higher self-efficacious beliefs are more likely to accept the app and show commitment even when faced with obstacles.
Individual stage of change	Users show different levels of open-mindedness regarding the use of a new app. Knowing their stage of change with respect to the apps facilitates appropriate implementation steps for them to become qualified, enthusiastic, and sustainable users [e.g., pre-contemplation, contemplation, preparation, and action and maintenance; (17)].
Individual identification with organization^*^	Refers to how individuals comprehend the organization and their relationship and level of engagement with the organization. These features may influence the willingness of employees (or individuals associated with the particular organization) to fully engage in implementation efforts or to take advantage of the app.
Other personal attributes	Further attributes that favor a successful implementation of apps are tolerance of ambiguity, intellectual ability, motivation, values, competence, performance, innovative ability, or learning style.
**Process**	
Planning	Apps should be implemented according to plan. Plans developed in advance should take the following points into account: Stakeholder needs and perspectives; strategies are tailored to appropriate subgroups (e.g., for hard-to-reach target groups); appropriate style, visual language, and metaphors are identified and used to deliver information and training; appropriate communication channels are identified and used; improvement toward goals and milestones is tracked with thorough monitoring and evaluation methods; and strategies (e.g., “dry runs” for testing) are used to accelerate use.
Engagement	Attracting and involving appropriate people in the implementation and use of the app through a combined strategy of social marketing, education, role modeling, training, and other similar activities. Important target groups can be e.g.,: (1) Opinion leaders: Persons with influence on the attitudes and
	beliefs of other users regarding the use of the app; (2) Champions: Persons who are dedicated to support and marketing, overcoming indifference or resistance that the app may provoke in the population; (3) External change agents: Persons who are connected to an external entity that formally influences or facilitates implementation-related decisions.
Execution	Execution and completion of the implementation according to a previously defined plan.
Reflection and evaluation	Quantitative and qualitative feedback on the progress and quality of implementation, accompanied by experience (e.g., from user reviews). Can be used to further improve the app (e.g., using quality improvement approaches). Clear and measurable evaluation objectives should be developed.

* *Might only be relevant for apps with a clear connection to an organization or organizational unit*.

## Discussion

Special attention should be paid to the successful implementation of health apps to warrant high quality care and service provision. Theories and frameworks may prove useful to systematically understand and assess implementation. The proposed adaptation of the CFIR might help to identify potentially relevant elements for a successful implementation, thus, providing a means to strengthen the future implementation and dissemination of health apps and DIPH in general. The described and adapted constructs provide a useful and comprehensive theoretical basis for understanding the implementation of health apps, though other theories, models, or frameworks might also guide such a process.

A narrative review by Nilsen (19) identified various implementation theories, models, and frameworks, of which the CFIR is one of the determinant frameworks (i.e., defining types of determinants that act as barriers or enablers and influence implementation outcomes). It is based on a synthesis of existing frameworks from different disciplines and therefore, provides a good overview of possible determinants for successful implementation. Besides such frameworks, process models (i.e., describing and/or guiding the process of translating research into practice), classic theories (i.e., theories that originate from fields external to implementation science), implementation theories (i.e., theories to provide understanding and/or explanation of aspects of implementation) and evaluation frameworks (i.e., concepts that specify aspects of the implementation that could be evaluated to ascertain its' success) represent theoretical approaches in implementation science. Clearly, choosing one of the theories or frameworks strongly depends on the focus and aim of the study which will be conducted. Some researchers and practitioners alike might find it useful to combine different theories or frameworks or even single aspects of such theories and frameworks, depending on the given context, to better understand and facilitate implementation.

This might also be the case when adapting the CFIR for research on implementing health apps. While the CFIR can be seen as one facilitating step to aid health app implementation, it might benefit from additional constructs that relate to digital concepts. One example which might complement the CFIR in a meaningful way is the Technology Acceptance Model [TAM; (20)]. The TAM aims at exploring the use of technology as a function of usability and ease of use, two central constructs in the interaction of humans and technology. Furthermore, an understanding of health apps as natural complex interventions due to their usually large amounts of interactions and varying intervention components might further ease the understanding of implementation requirements for health apps to avoid implementation failure (2). Additionally, technical aspects for clinical validation also should be considered. Such aspects might be drawn from frameworks for biometric monitoring technologies (21, 22) which highlight the need for appropriate vocabulary and standardized approaches to evaluate digitally measured biomarkers, including defining performance characteristics and acceptance criteria. Furthermore, the adaptation of the CFIR for health apps might also supplement existing guidelines when focusing on implementation of apps the comply with guidelines (e.g., Xcertia Guidelines) (23).

As a perspective article, the aim of the current piece is to present a viewpoint on using the CFIR as a potential support for implementing health apps (e.g., for public health purpose). As such, the article focuses on the CFIR as one of the principal implementation frameworks and how it might be adopted for future studies on health app implementation. Therefore, future studies on implementation frameworks for health apps should consider conducting comparative analyses about different implementation frameworks, also including the presented adaptation of the CFIR (e.g., *via* systematic reviews) while other studies should investigate how useful and appropriate the adaptations to the CFIR are and might modify it to their needs. One important aspect is likely to be the domain of cyber security, currently not part of this article, given the origin of the CFIR in non-digital implementation. Considering cyber security as a cross-cutting issue for several constructs (e.g., to increase trust in the app) is a highly relevant topic for ongoing adaptations.

Another outlook is to apply the adapted CFIR to our own and other's current and future research about digital health apps from a public health perspective. Other fields for application (e.g., health psychology) might find our framework helpful as well. In the meantime, the adjusted CFIR will be presented on conferences and disseminated through our networks with the aim to gain additional feedback from different perspectives (e.g., HCI) to further develop it. Such activities will take place under the umbrella of the Leibniz ScienceCampus Digital Public Health (LSC DiPH) (https://www.lsc-digital-public-health.de/en/). The LSC DiPH aims to develop a much-needed public health perspective on evidence-based novel public health, ethical, sociocultural, and equity-related challenges.

## Conclusion

Implementation researchers should assess each construct for its properties, carefully tailor and operationalize the definitions for their study (paying particular attention to the sometimes imprecise boundaries between constructs), and identify the level(s) at which each construct should be assessed and defined. They must also decide how and when to measure and assess, taking into account the transient nature of the state of each of these contextual factors. Each choice and rationale should be documented along with the results related to each construct (13). Using the CFIR and its definition of domains and related constructs might foster clarity regarding implementation effort and comparability with other implementation studies.

## Data Availability Statement

The original contributions generated for the study are included in the article/Supplementary Material, further inquiries can be directed to the corresponding author/s.

## Author Contributions

JW drafted the manuscript with substantial input by HZ. All authors contributed to the article and approved the submitted version.

## Conflict of Interest

The authors declare that the research was conducted in the absence of any commercial or financial relationships that could be construed as a potential conflict of interest.
