# Supplementary material for: Implementing Health Apps for Digital Public Health – An Implementation Science Approach Adopting the Consolidated Framework for Implementation Research
Source: Front Public Health. 2021 May 7;9:610237. doi: 10.3389/fpubh.2021.610237 (PMC8137849; doi:10.3389/fpubh.2021.610237)
Supplement: Supplementary file 1 [file Table_1.DOCX]

***Supplement 1***

**“Implementing health apps for Digital Public Health”**

| **Original Construct [1]** | **Original Construct Description [1]** | **Adapted Construct** | **Adapted Construct Description** |
| --- | --- | --- | --- |
| *Intervention characteristics* |  | *Features of the App* |  |
| Intervention Source | Perception of key stakeholders about whether the intervention is externally or internally developed [2]. An intervention may be internally developed as a good idea, solution to a problem, or other grass-roots effort, or may be developed by an external entity (*e.g*., vendor or research group) [2]. The legitimacy of the source may also influence implementation. | Source of the app | Clear and transparent description of the app, its functions and sources of the source code (e.g. developer). Preferentially, the source code should be freely available. Clear and transparent description of data usage and data protection. |
| Evidence strength and quality | Stakeholders' perceptions of the quality and validity of evidence supporting the belief that the intervention will have desired outcomes. Sources of evidence may include published literature, guidelines, anecdotal stories from colleagues, information from a competitor, patient experiences, results from a local pilot, and other sources [3, 4]. | Evidence strength and quality | Description of the current status of previous, scientific findings on the quality and validity of the app. |
| Relative advantage | Stakeholders' perception of the advantage of implementing the intervention versus an alternative solution [5]. | Relative advantage | Demonstration of the positive benefit of the stakeholder's use of the app. |
| Adaptability | The degree to which an intervention can be adapted, tailored, refined, or reinvented to meet local needs. Adaptability relies on a definition of the 'core components' (the essential and indispensible elements of the intervention itself) versus the 'adaptable periphery' (adaptable elements, structures, and systems related to the intervention and organization into which it is being implemented) of the intervention [2, 6], as described in the Overview section. A component analysis can be performed to identify the core versus adaptable periphery components [7], but often the distinction is one that can only be discerned through trial and error over time as the intervention is disseminated more widely and adapted for a variety of contexts [8]. The tension between the need to achieve full and consistent implementation across multiple contexts while providing the flexibility for local sites to adapt the intervention as needed is real and must be balanced, which is no small challenge [9]. | Adaptability | Description of the extent to which core components of an app can be adapted, tailored, refined or newly developed to individual or local needs (e.g., in the event of different legal regulations or low network coverage). |
| Trialability | The ability to test the intervention on a small scale in the organization [2], and to be able to reverse course (undo implementation) if warranted [10]. The ability to trial is a key feature of the plan-do-study-act quality improvement cycle that allows users to find ways to increase coordination to manage interdependence [11]. Piloting allows individuals and groups to build experience and expertise, and time to reflect upon and test the intervention [12], and usability testing (with staff and patients) promotes successful adaptation of the intervention [10]. | Feasibility | The ability to test the app on a small scale under real conditions (e.g., in the course of a pilot study) and to allow a reversal of the implementation when justified (e.g., when the app is no longer needed or superseded by other developments) |
| Complexity | Perceived difficulty of implementation, reflected by duration, scope, radicalness, disruptiveness, centrality, and intricacy and number of steps required to implement [2, 13]. Radical interventions require significant reorientation and non-routine processes to produce fundamental changes in the organization's activities and reflects a clear departure from existing practices [2]. One way to determine complexity is by assessing 'length' (the number of sequential sub-processes or steps for using or implementing an intervention) and 'breadth' (number of choices presented at decision points) [14]. Complexity is also increased with higher numbers of potential target organizational units (teams, clinics, departments) or types of people (providers, patients, managers) targeted by the intervention [14], and the degree to which the intervention will alter central work processes [13]. | Complexity (practical implementation difficulties) | Perceived implementation difficulties which are reflected in duration, scope, disruptivity, centrality, complexity and number of steps required for implementation. Increased complexity may be accompanied by increased implementation effort (e.g., due to growing need for content coordination). |
| Design quality and packaging | Perceived excellence in how the intervention is bundled, presented, and assembled [15]. | Design quality and packaging | The app must be able to meet common design requirements (e.g., verified by design tests). |
| Cost | Costs of the intervention and costs associated with implementing that intervention, including investment, supply, and opportunity costs. It is important to differentiate this construct from available resources (part of inner setting, below). In many contexts, costs are difficult to capture and available resources may have a more direct influence on implementation. | Costs | Costs of the app and costs related to the implementation of this app, including investment, supply and opportunity costs. Costs should be in relation to the expected benefits, both for the user and the developer. |
| *Outer Setting* |  | *Outer Setting* |  |
| Patient needs and resources | The extent to which patient needs, as well as barriers and facilitators to meet those needs, are accurately known and prioritized by the organization. Clearly, improving the health and well-being of patients is the mission of all healthcare entities, and many calls have gone out for organizations to be more patient centered [16]. Patient-centered organizations are more likely to implement change effectively [17]. Many theories of research uptake or implementation acknowledge the importance of accounting for patient characteristics [10, 12, 18], and consideration of patients needs and resources must be integral to any implementation that seeks to improve patient outcomes [16]. The Practical, Robust Implementation and Sustainability Model PRISM delineates six elements that can help guide evaluation of the extent to which patients are at the center of organizational processes and decisions: patient choices are provided, patient barriers are addressed, transition between program elements is seamless, complexity and costs are minimized, and patients have high satisfaction with service and degree of access and receive feedback [10]. | User needs and resources | Consideration of the needs and resources of the app's target group (e.g., perceived vulnerability, literacy, language, but also storage space, data volume, availability of hardware, network connection) |
| Cosmopolitanism | The degree to which an organization is networked with other external organizations. Organizations that support and promote external boundary-spanning roles of their staff are more likely to implement new practices quickly [2]. The collective networks of relationships of individuals in an organization represent the social capital of the organization [19]. Social capital is one term used to describe the quality and the extent of those relationships and includes dimensions of shared vision and information sharing. One component of social capital is external bridging between people or groups outside the organization [2]. | Cosmopolitanism | Cooperation and exchange with external developers and stakeholders (e.g., professional associations) |
| Peer pressure | Mimetic or competitive pressure to implement an intervention, typically because most or other key peer or competing organizations have already implemented or in pursuit of a competitive edge. 'Peers' can refer to any outside entity with which the organization feels some degree of affinity or competition at some level within their organization (*e.g*., competitors in the market, other hospitals in a network). The pressure to implement can be particularly strong for late-adopting organizations [20]. | Peer pressure | Other providers are already present on the market with apps, while new providers still have to follow suit. The resulting "peer pressure" can make apps more error-prone. |
|  | Broad constructs that encompass external strategies to spread interventions, including policy and regulations (governmental or other central entity), external mandates, recommendations and guidelines, pay-for-performance, collaboratives, and public or benchmark reporting [8]. | External policies and incentives | Legal regulations, recommendations or guidelines for the use of the app. |
| *Inner Setting* |  | *Inner Setting** |  |
| Structural characteristics | The social architecture, age, maturity, and size of an organization. Social architecture describes how large numbers of people are clustered into smaller groups and differentiated, and how the independent actions of these differentiated groups are coordinated to produce a holistic product or service [21]. Structural characteristics are, by-and-large, quantitative measures and, in most cases, measurement instruments and approaches have been developed for them. Damenpour conducted a meta-analysis of many structural determinants based on 23 studies conducted outside the healthcare sector [22]. Functional differentiation is the internal division of labor where coalitions of professionals are formed into differentiated units. The number of units or departments represents diversity of knowledge in an organization. The more stable teams are (members are able to remain with the team for an adequate period of time; low turnover), the more likely implementation will be successful [23]. Administrative intensity (the ratio of managers to total employees) is positively associated with innovation [22]. Centralization (the concentration of decision-making autonomy) has been shown to be negatively associated with innovation [22], but has also been found to be positive or negatively associated, depending on the stage of intervention (initiative stage versus implementation stage) [24]. Size, age, maturity, and degree of specialization (the uniqueness of the niche or market for the organization's products or services) also influence implementation [2]. | Structural characteristics | A stable team, which is entrusted with the development of the app, increases the probability of a successful implementation of the app. Centralization of decision-making autonomy can be negatively associated with innovation. Administrative intensity may be positively associated with innovation. Size, age, maturity and degree of specialization of the developer (the uniqueness of the niche or market for the organization's products or services) also influence implementation. |
| Networks and communication | The nature and quality of webs of social networks and the nature and quality of formal and informal communications within an organization. Research on organizational change has moved beyond reductionist measures of organizational structure, and increasingly embraces the complex role that networks and communications have on implementation of change interventions [25]. Connections between individuals, units, services, and hierarchies may be strong or weak, formal or informal, tangible or intangible. Social capital describes the quality and the extent of relationships and includes dimensions of shared vision and information sharing. One component of social capital is the internal bonding of individuals within the same organization [2]. Complexity theory posits that relationships between individuals may be more important than individual attributes [26], and building these relationships can positively influence implementation [27]. These relationships may manifest to build a sense of 'teamness' or 'community' that may contribute to implementation effectiveness [23].  Regardless of how an organization is structurally organized, the importance of communication across the organization is clear. Communication failures are involved with the majority of sentinel events in US hospitals [28]. High quality of formal communications contributes to effective implementation [29]. Making staff feel welcome (good assimilation), peer collaboration and open feedback and review among peers and across hierarchical levels, clear communication of mission and goals, and cohesion between staff and informal communication quality, all contribute to effective implementation [29]. | Networks and communications | High social capital within a team, i.e. dimensions of shared vision and information sharing, can contribute to effective implementation through a sense of "team spirit" or "community". |
| Culture | Norms, values, and basic assumptions of a given organization [30]. Most change efforts are targeted at visible, mostly objective, aspects of an organization that include work tasks, structures, and behaviors. One explanation for why so many of these initiatives fail centers on the failure to change less tangible organizational assumptions, thinking, or culture [31].  Some researchers have a relatively narrow definition of culture, while other researchers incorporate nearly every construct related to inner setting. In the next section we highlight the concept of 'climate.' As with 'culture,' climate suffers from inconsistent definition. Culture and climate can, at times, be interchangeable across studies, depending on the definition used [32]. A recent review found 54 different definitions for organizational climate [30] and, likewise, many definitions exist for culture [32]. Culture is often viewed as relatively stable, socially constructed, and subconscious [32]. The CFIR embraces this latter view and differentiates climate as the localized and more tangible manifestation of the largely intangible, overarching culture [30]. Climate is a phenomenon that can vary across teams or units, and is typically less stable over time compared to culture. | Culture | Norms and values, or a mindset and culture of an app provider. |
| Implementation climate | The absorptive capacity for change, shared receptivity of involved individuals to an intervention [2], and the extent to which use of that intervention will be 'rewarded, supported, and expected within their organization' [33]. Climate can be assessed through tangible and relatively accessible means such as policies, procedures, and reward systems [30]. Six sub-constructs contribute to a positive implementation climate for an intervention: tension for change, compatibility, relative priority, organizational incentives and rewards, goals and feedback, and learning climate.   1. Tension for change: The degree to which stakeholders perceive the current situation as intolerable or needing change [2, 29]. 2. Compatibility: The degree of tangible fit between meaning and values attached to the intervention by involved individuals, how those align with individuals' own norms, values, and perceived risks and needs, and how the intervention fits with existing workflows and systems [2, 33]. The more individuals perceive alignment between the meaning they attach to the intervention and meaning communicated by upper management, the more effective implementation is likely to be. For example, providers may perceive an intervention as a threat to their autonomy, while leadership is motivated by the promise of better patient outcomes. 3. Relative priority: Individuals' shared perception of the importance of the implementation within the organization [10, 15, 33]. 4. Organizational incentives and rewards: Extrinsic incentives such as goal-sharing awards, performance reviews, promotions, and raises in salary, as well as less tangible incentives such as increased stature or respect [15, 34]. 5. Goals and feedback: The degree to which goals are clearly communicated, acted upon, and fed back to staff and alignment of that feedback with goals [14, 29, 35]. The Chronic Care Model emphasizes the importance of relying on multiple methods of evaluation and feedback including clinical, performance, and economic evaluations and experience [36]. 6. Learning climate: A climate in which: leaders express their own fallibility and need for team members' assistance and input; team members feel that they are essential, valued, and knowledgeable partners in the change process; individuals feel psychologically safe to try new methods; and there is sufficient time and space for reflective thinking and evaluation (in general, not just in a single implementation) [15, 33, 37]. These interrelated practices and beliefs support and enable employee and organizational skill development, learning, and growth to maximize an organization's absorptive capacity for new knowledge and methods [2]. Quantitative measurement instruments are available for measuring an organization's 'learning' capability [38].   Readiness for implementation: Tangible and immediate indicators of organizational commitment to its decision to implement an intervention, consisting of three sub-constructs (leadership engagement, available resources, and access to information and knowledge). Implementation readiness is differentiated from implementation climate in the literature by its inclusion of specific tangible and immediate indicators of organizational commitment to its decision to implement an intervention.   1. Leadership engagement: Commitment, involvement, and accountability of leaders and managers [15, 35] with the implementation. The term 'leadership' can refer to leaders at any level of the organization, including executive leaders, middle management, front-line supervisors, and team leaders, who have a direct or indirect influence on the implementation. One important dimension of organizational commitment is managerial patience (taking a long-term view rather than short-term) to allow time for the often inevitable reduction in productivity until the intervention takes hold [15]. 2. Available resources: The level of resources dedicated for implementation and ongoing operations including money, training, education, physical space, and time [2, 5, 23, 29, 39, 40]. 3. Access to information and knowledge: Ease of access to digestible information and knowledge about the intervention and how to incorporate it into work tasks [2]. Information and knowledge includes all sources such as experts, other experienced staff, training, documentation, and computerized information systems. | Implementation climate | The capacity to absorb change, the common receptivity of the people involved with an app and the extent to which the use of that app "is rewarded, supported and expected within their organization". Six substructures contribute to a positive climate for the implementation of an app: Excitement for change, compatibility, relative priority, organizational incentives and rewards, goals and feedback and learning climate.  Readiness to implement: Concrete and immediate indicators of the organization's commitment to its decision to implement an app, including commitment of leadership, available resources and access to information and knowledge. |
| *Characteristics of individuals* |  | *Characteristics of individuals* |  |
| Knowledge and beliefs about the intervention | Individuals' attitudes toward and value placed on the intervention, as well as familiarity with facts, truths, and principles related to the intervention. Skill in using the intervention is a primarily cognitive function that relies on adequate how-to knowledge and knowledge of underlying principles or rationale for adopting the intervention [41]. Enthusiastic use of an intervention can be reflected by a positive affective response to the intervention. Often, subjective opinions obtained from peers based on personal experiences are more accessible and convincing, and these opinions help to generate enthusiasm [41]. Of course, the converse is true as well, often creating a negative source of active or passive resistance [42]. The degree to which new behaviors are positively or negatively valued heightens intention to change, which is a precursor to actual change [43]. | Knowledge and convictions about the app | Perception of a credible external presentation of the app, as well as knowledge of the users how to handle it. |
| Self-efficacy | Individual belief in their own capabilities to execute courses of action to achieve implementation goals [44]. Self-efficacy is a significant component in most individual behavior change theories [45]. Self-efficacy is dependent on the ability to perform specific actions within a specific context. The more confident an individual feels about his or her ability to make the changes needed to achieve implementation goals, the higher their self-efficacy. Individuals with high self-efficacy are more likely to make a decision to embrace the intervention and exhibit committed use even in the face of obstacles. | Self-efficacy | The more confident persons feel about their ability to use the app effectively, the higher their self-efficacy. People with high self-efficacy are more likely to accept the app and show commitment even when faced with obstacles. |
| Individual stage of change | Characterization of the phase an individual is in, as he or she progresses toward skilled, enthusiastic, and sustained use of the intervention [13, 15]. The specific stages used will depend on the underlying model being used in the study. Prochaska's trans-theoretical model characterizes these stages as pre-contemplation, contemplation, preparation, and action and maintenance [46]. Rogers' diffusion theory delineates five stages [41]. Grol *et al*. describe a five-stage model with ten sub-stages based on their synthesis of the literature [13]. | Individual stage of change | Users show different levels of open-mindedness regarding the use of a new app. Knowing their stage of change with respect to the apps facilitates appropriate implementation steps for them to become qualified, enthusiastic and sustainable users (e.g., pre-contemplation, contemplation, preparation, and action and maintenance; 15). |
| Individual identification with organization | A broad construct related to how individuals perceive the organization and their relationship and degree of commitment to that organization. These attributes may affect the willingness of staff to fully engage in implementation efforts or use the intervention [47, 48]. These measures have been studied very little in healthcare, but may be especially important when evaluating the influence of implementation leaders' (described under Process below) on implementation efforts. Organizational citizenship behavior characterizes how well organizational identity is taken on by individuals and whether, because they associate themselves with the organization, they are willing to put in extra effort, talk well of the organization, and take risks in their organization [49, 50]. Organizational justice is an individual's perception of distributive and procedural fairness in the organization [47]. Emotional exhaustion is an ongoing state of emotional and physical depletion or burnout [51], and may negatively influence implementation by stunting the ability and energy of an individual to help or initiate change [52]. The Agency for Healthcare Research and Quality recently published a guide for determining whether a particular implementation will be successful that includes questions about individual perceptions of whether they believe the organization could be doing a better job, belief about whether work is done efficiently, and whether there are inequities as potential barriers to implementation [53]. The organizational social context measure, developed by Glisson *et al*., includes constructs related to psychological climate (perception of the psychological influence of work environment) and work attitudes (job satisfaction and organizational commitment) [54]. | Individual identification with organization* | Refers to how individuals perceive the organization and their relationship and level of engagement with the organization. These characteristics can affect the willingness of employees (or persons associated with the organization at hand) to become fully involved in the implementation effort or to take advantage of the app. |
| Other personal attribute | This is a broad construct to include other personal traits. Traits such as tolerance of ambiguity, intellectual ability, motivation, values, competence, capacity, innovativeness [55], tenure [55], and learning style have not received adequate attention by implementation researchers [2]. | Other personal attributes | Further attributes that favor a successful implementation of apps are tolerance of ambiguity, intellectual ability, motivation, values, competence, performance, innovative ability or learning style. |
| *Process* |  | *Process* |  |
| Planning | The degree to which a scheme or method of behavior and tasks for implementing an intervention are developed in advance and the quality of those schemes or methods. The fundamental objective of planning is to design a course of action to promote effective implementation by building local capacity for using the intervention, collectively and individually [8]. The specific steps in plans will be based on the underlying theories or models used to promote change at organization and individual levels [13]. For example, the Institute for Healthcare Improvement [56, 57], Grol *et al*. [58], and Glisson and Schoenwald [59] all describe comprehensive approaches to implementation on which implementation plans can be developed. However, these theories prescribe different sets of activities because they were developed in different contexts–though commonalities exist as well. Grol *et al*. list 14 different bodies of theories for changing behaviors in social or organizational contexts [13], and Estabrooks *et al*. list 18 different models of organizational innovation [60]. Thus, the particular content of plans will vary depending on the theory or model being used to guide implementation. Implementation plans can be evaluated by the degree to which five considerations guide planning: stakeholders' needs and perspectives are considered; strategies are tailored for appropriate subgroups (*e.g*., delineated by professional, demographic, cultural, organizational attributes); appropriate style, imagery, and metaphors are identified and used for delivering information and education; appropriate communication channels are identified and used; progress toward goals and milestones is tracked using rigorous monitoring and evaluation methods [2, 41]; and strategies are used to simplify execution. The latter step may include plans for dry runs (simulations or practice sessions) to allow team members to learn how to use the intervention before going live [23], running trials to allow users to test procedures, gain confidence, and build an environment of psychological safety [23], or taking an incremental approach that breaks the intervention down into manageable parts that can be implemented incrementally [22]. The plan can be formal or informal but should consider all salient contextual factors–both modifiable and non-modifiable. Workarounds can be developed for non-modifiable factors, and strategies can be designed to change factors that can be modified (*e.g*., increase stakeholders' knowledge of the intervention). | Planning | Apps should be implemented according to plan. Plans developed in advance should take the following points into account: Stakeholder needs and perspectives; strategies are tailored to appropriate subgroups (e.g., for hard-to-reach target groups); appropriate style, visual language and metaphors are identified and used for information and education provision; appropriate communication channels are identified and used; progress towards goals and milestones is tracked using rigorous monitoring and evaluation methods; and strategies (e.g., “dry runs” for piloting) are used to facilitate use. |
| Engaging | Attracting and involving appropriate individuals in the implementation and use of the intervention through a combined strategy of social marketing, education, role modeling, training, and other similar activities. Engaging members of teams tasked with implementing an intervention (or to be 'first users') is an often overlooked part of implementation [61]. It is vital that early members are carefully and thoughtfully selected or allowed to rise naturally [23, 61], especially 'implementation leaders' and 'champions.' If early users and leaders are homophilous (similar socioeconomic, professional, educational, and cultural backgrounds) with intended users, individuals will be more likely to adopt the intervention [2]. The influence of these leaders can be evaluated by assessing their presence or absence (*e.g*., does the implementation effort have a clear champion or not?), how they are brought on board (*e.g*., appointed, volunteered), their role in the organization (formal and/or informal roles), and their role in implementation. One means by which influence is transmitted is role modeling [62]. We have identified four types of implementation leaders. Terms and definitions of roles vary widely in the literature. The remainder of this section suggests standard definitions for each:   1. Opinion leaders: Individuals in an organization who have formal or informal influence on the attitudes and beliefs of their colleagues with respect to implementing the intervention [2, 41]. There is general agreement that there are two different types of opinion leaders, experts and peers. Expert opinion leaders exert influence through their authority and status [2]. Peer opinion leaders exert influence through their representativeness and credibility [2]. 2. Formally appointed internal implementation leaders: Individuals from within the organization who have been formally appointed with responsibility for implementing an intervention as coordinator, project manager, team leader, or other similar role. These leaders may or may not have explicit time dedicated to the task. Implementation is 'part of the job.' 3. Champions: 'Individuals who dedicate themselves to supporting, marketing, and 'driving through an [implementation]' [63], overcoming indifference or resistance that the intervention may provoke in an organization. A defining characteristic of champions is their willingness to risk informal status and reputation because they believe so strongly in the intervention [64]. The main distinction of champions from opinion leaders is that champions actively associate themselves with support of the intervention during implementation. There is the old adage that an intervention 'either finds a champion or dies' [65]. 4. External change agents: Individuals who are affiliated with an outside entity who formally influence or facilitate intervention decisions in a desirable direction. They usually have professional training in a technical field related to organizational change science or in the technology being introduced into the organization. This role includes outside researchers who may be implementing a multi-site intervention study and other formally appointed individuals from an external entity (related or unrelated to the organization); *e.g*., a facilitator from a corporate or regional office or a hired consultant. | Engagement | Attracting and involving appropriate people in the implementation and use of the app through a combined strategy of social marketing, education, role modelling, training and other similar activities. Important target groups can be e.g.: 1) Opinion leaders: Persons with influence on the attitudes and beliefs of other users regarding the use of the app; 2) Champions: Persons who are dedicated to support and marketing, overcoming indifference or resistance that the app may provoke in the population; 3) External change agents: Persons who are connected to an external entity that formally influences or facilitates implementation-related decisions. |
| Executing | Carrying out or accomplishing the implementation according to plan. Execution of an implementation plan may be organic with no obvious or formal planning, which makes execution difficult to assess. Quality of execution may consist of the degree of fidelity of implementation to planned courses of action [7], intensity (quality and depth) of implementation [66], timeliness of task completion, and degree of engagement of key involved individuals (*e.g*., implementation leaders) in the implementation process. | Execution | Execution and completion of the implementation according to a previously defined plan. |
| Reflecting and evaluating | Quantitative and qualitative feedback about the progress and quality of implementation accompanied with regular personal and team debriefing about progress and experience. It is important to differentiate this processual construct from the Goals and Feedback construct under Inner Setting, described above. The focus here is specifically related to implementation efforts. Evaluation includes traditional forms of feedback, such as reports, graphs, and qualitative feedback and anecdotal stories of success [45]. Objectives should be specific, measurable, attainable, relevant, and timely (the SMART rubric) [53]. Less attention is paid in the literature to the need for, and value of, group and personal reflection. Dedicating time for reflecting or debriefing before, during, and after implementation is one way to promote shared learning and improvements along the way [23]. | Reflection and evaluation | Quantitative and qualitative feedback on the progress and quality of implementation, accompanied by experience (e.g., from user reviews). Can be used to further improve the app (e.g., using quality improvement approaches). Clear and measurable evaluation objectives should be developed. |

Note: *might only be relevant for apps with a clear connection to an organization or organizational unit.

**References**

1. Damschroder LJ, Aron DC, Keith RE, et al: Fostering implementation of health services research findings into practice: a consolidated framework for advancing implementation science. Implement Sci. 2009, 4:50. doi: 10.1186/1748-5908-4-50
2. Greenhalgh T, Robert G, Macfarlane F, Bate P, Kyriakidou O: Diffusion of innovations in service organizations: systematic review and recommendations. Milbank Q. 2004, 82: 581-629. 10.1111/j.0887-378X.2004.00325.x.
3. Rycroft-Malone J, Harvey G, Kitson A, McCormack B, Seers K, Titchen A: Getting evidence into practice: ingredients for change. Nurs Stand. 2002, 16: 38-43.
4. Stetler CB: Updating the Stetler Model of research utilization to facilitate evidence-based practice. Nurs Outlook. 2001, 49: 272-279. 10.1067/mno.2001.120517.
5. Gustafson DH, Sainfort F, Eichler M, Adams L, Bisognano M, Steudel H: Developing and testing a model to predict outcomes of organizational change. Health Serv Res. 2003, 38: 751-776. 10.1111/1475-6773.00143.
6. Fixsen DL, Naoom SF, Blase KA, Friedman RM, Wallace F: Implementation Research: A Synthesis of the Literature. Book Implementation Research: A Synthesis of the Literature (Editor ed.^eds.). 2005, City: University of South Florida, Louis de la Parte Florida Mental Health Institute
7. Carroll C, Patterson M, Wood S, Booth A, Rick J, Balain S: A conceptual framework for implementation fidelity. Implement Sci. 2007, 2: 40-10.1186/1748-5908-2-40.
8. Mendel P, Meredith LS, Schoenbaum M, Sherbourne CD, Wells KB: Interventions in organizational and community context: a framework for building evidence on dissemination and implementation in health services research. Adm Policy Ment Health. 2008, 35: 21-37. 10.1007/s10488-007-0144-9.
9. Perrin KM, Burke SG, O'Connor D, Walby G, Shippey C, Pitt S, McDermott RJ, Forthofer MS: Factors contributing to intervention fidelity in a multi-site chronic disease self-management program. Implement Sci. 2006, 1: 26-10.1186/1748-5908-1-26.
10. Feldstein AC, Glasgow RE: A practical, robust implementation and sustainability model (PRISM) for integrating research findings into practice. Joint Commission journal on quality and patient safety/Joint Commission Resources. 2008, 34: 228-243.
11. Leeman J, Baernholdt M, Sandelowski M: Developing a theory-based taxonomy of Methods for implementing change in practice. J Adv Nurs. 2007, 58: 191-200. 10.1111/j.1365-2648.2006.04207.x.
12. Rycroft-Malone JA, Kitson G, Harvey B, McCormack K, Seers AT, Estabrooks C: Ingredients for change: revisiting a conceptual framework. (Viewpoint). Quality and Safety in Health Care. 2002, 11: 174-180. 10.1136/qhc.11.2.174.
13. Grol RP, Bosch MC, Hulscher ME, Eccles MP, Wensing M: Planning and studying improvement in patient care: The use of theoretical perspectives. Milbank Q. 2007, 85: 93-138. 10.1111/j.1468-0009.2007.00478.x.
14. Kochevar LK, Yano EM: Understanding health care organization needs and context. Beyond performance gaps. J Gen Intern Med. 2006, 21 (Suppl 2): S25-29.
15. Klein KJ, Conn AB, Sorra JS: Implementing computerized technology: An organizational analysis. J Appl Psychol. 2001, 86: 811-824. 10.1037/0021-9010.86.5.811.
16. Institute of Medicine: Crossing the Quality Chasm: A New Health System for the 21st Century. 2001, Washington, DC: National Academy Press
17. Shortell SM, Marsteller JA, Lin M, Pearson ML, Wu SY, Mendel P, Cretin S, Rosen M: The role of perceived team effectiveness in improving chronic illness care. Med Care. 2004, 42: 1040-1048. 10.1097/00005650-200411000-00002.
18. Graham ID, Logan J: Innovations in knowledge transfer and continuity of care. Can J Nurs Res. 2004, 36: 89-103.
19. Brehem J, Rahn W: Individual Level Evidence for the Causes and Consequences of Social Capital. American Journal of Political Science. 1997, 41: 999-1023. 10.2307/2111684.
20. Walston SL, Kimberly JR, Burns LR: Institutional and economic influences on the adoption and extensiveness of managerial innovation in hospitals: The case of reengineering. Med Care Res Rev. 2001, 58: 194-228. 10.1177/107755870105800203. discussion 229-133
21. Thompson J, Scott W, Zald M: Organizations in Action: Social Science Bases of Administrative Theory. 2003, Edison, NJ: Transaction Publishers
22. Damanpour F: Organizational Innovation: A Meta-Analysis of Effects of Determinants and Moderators. The Academy of Management Journal. 1991, 34: 555-590. 10.2307/256406.
23. Edmondson AC, Bohmer RM, Pisana GP: Disrupted routines: Team learning and new technology implementation in hospitals. Adm Sci Q. 2001, 46: 685-716. 10.2307/3094828.
24. Dewar RD, Dutton JE: The Adoption of Radical and Incremental Innovations: An Empirical Analysis. Management Science. 1986, 32: 1422-1433. 10.1287/mnsc.32.11.1422.
25. Fitzgerald LA, van Eijnatten FM: Reflections: Chaos in organizational change. Journal of Organizational Change Management. 2002, 15: 402-411. 10.1108/09534810210433700.
26. Plsek PE, Wilson T: Complexity, leadership, and management in healthcare organisations. BMJ. 2001, 323: 746-749. 10.1136/bmj.323.7313.625.
27. Safran DG, Miller W, Beckman H: Organizational dimensions of relationship-centered care. Theory, evidence, and practice. J Gen Intern Med. 2006, 21 (Suppl 1): S9-15. 10.1111/j.1525-1497.2006.00303.x.
28. Pronovost PJ, Berenholtz SM, Goeschel CA, Needham DM, Sexton JB, Thompson DA, Lubomski LH, Marsteller JA, Makary MA, Hunt E: Creating high reliability in health care organizations. Health Serv Res. 2006, 41: 1599-1617. 10.1111/j.1475-6773.2006.00567.x.
29. Simpson DD, Dansereau DF: Assessing Organizational Functioning as a Step Toward Innovation. NIDA Science & Practice Perspectives. 2007, 3: 20-28.
30. Gershon R, Stone PW, Bakken S, Larson E: Measurement of Organizational Culture and Climate in Healthcare. J Nurs Adm. 2004, 34: 33-40. 10.1097/00005110-200401000-00008.
31. van Eijnatten FM, Galen M: Chaos, dialogue and the dolphin's strategy. Journal of Organizational Change Management. 2002, 15: 391-401. 10.1108/09534810210433692.
32. Martin J: Organizational culture: Mapping the terrain. 2002, Thousand Oaks, CA: Sage Publications
33. Klein KJ, Sorra JS: The Challenge of Innovation Implementation. The Academy of Management Review. 1996, 21: 1055-1080. 10.2307/259164.
34. Helfrich CD, Weiner BJ, McKinney MM, Minasian L: Determinants of implementation effectiveness: adapting a framework for complex innovations. Med Care Res Rev. 2007, 64: 279-303. 10.1177/1077558707299887.
35. VanDeusen Lukas CV, Holmes SK, Cohen AB, Restuccia J, Cramer IE, Shwartz M, Charns MP: Transformational change in health care systems: An organizational model. Health Care Manage Rev. 2007, 32: 309-320.
36. Wagner EH, Austin BT, Davis C, Hindmarsh M, Schaefer J, Bonomi A: Improving chronic illness care: translating evidence into action. Health Aff (Millwood). 2001, 20: 64-78. 10.1377/hlthaff.20.6.64.
37. Nembhard I, Edmonson A: Making it safe: the effects of leader inclusiveness and professional status on psychological safety and improvement efforts in health care teams. Journal of Organizational Behavior. 2006, 27: 941-966. 10.1002/job.413.
38. Templeton GF, Lewis BR, Snyder CA: Development of a measure for the organizational learning construct. Journal of Management Information Systems. 2002, 19: 175-218.
39. Fitzgerald LE, Wood FM, Hawkins C: Interlocking Interactions: the Diffusion of Innovations in Health Care. Human Relations. 2002, 55: 1429-1449. 10.1177/001872602128782213.
40. Weiner BJ, Savitz LA, Bernard S, Pucci LG: How do integrated delivery systems adopt and implement clinical information systems?. Health Care Manage Rev. 2004, 29:51.
41. Rogers E: Diffusion of Innovations. 2003, New York, NY: Free Press, 5
42. Saint S, Kowalski CP, Banaszak-Holl J, Forman J, Damschroder L, Krein SL: How Active Resisters and Organizational Constipators Affect Health Care-Acquired Infection Prevention Efforts. The Joint Commission Journal on Quality and Patient Safety. 2009, 35: 239-246.
43. Ajzen I: The theory of planned behavior. Organ Behav Hum Decis Process. 1991, 50: 179-211. 10.1016/0749-5978(91)90020-T.
44. Bandura A: Self-efficacy: toward a unifying theory of behavioral change. Psychol Rev. 1977, 84: 191-215. 10.1037/0033-295X.84.2.191.
45. National Institutes of Health: Theory at a Glance: A guide for health promotion practice. Bethesda : National Institutes of Health; 2005.
46. Prochaska JO, Velicer WF: The transtheoretical model of health behavior change. Am J Health Promot. 1997, 12: 38-48.
47. Greenberg J: Organizational justice: Yesterday, today, and tomorrow. Journal of Management. 1990, 16: 399-432. 10.1177/014920639001600208.
48. Abraham R: Organizational cynicism: bases and consequences. Genet Soc Gen Psychol Monogr. 2000, 126: 269-292.
49. Pearce CL, Ensley MD: A reciprocal and longitudinal investigation of the innovation process: the central role of shared vision in product and process innovation teams (PPITs). Journal of Organizational Behavior. 2004, 25: 259-278. 10.1002/job.235.
50. Smith AC, Organ D, Near J: Organizational Citizenship Behavior: Its Nature and Antecedents. J Appl Psychol. 1983, 68: 653-663. 10.1037/0021-9010.68.4.653.
51. Cropanzano R, Rupp DE, Byrne ZS: The relationship of emotional exhaustion to work attitudes, job performance, and organizational citizenship behaviors. J Appl Psychol. 2003, 88: 160-169. 10.1037/0021-9010.88.1.160.
52. Estabrooks CA, Midodzi WK, Cummings GG, Wallin L: Predicting research use in nursing organizations: a multilevel analysis. Nurs Res. 2007, 56: S7-23. 10.1097/01.NNR.0000280647.18806.98.
53. Brach C, Lenfestey N, Roussel A, Amoozegar J, Sorensen A: Will It Work Here? A Decisionmaker's Guide to Adopting Innovations. 2008, Agency for Healthcare Research & Quality (AHRQ).
54. Glisson C, Landsverk J, Schoenwald S, Kelleher K, Hoagwood KE, Mayberg S, Green P: Assessing the Organizational Social Context (OSC) of Mental Health Services: Implications for Research and Practice. Adm Policy Ment Health. 2008, 35: 98-113. 10.1007/s10488-007-0148-5.
55. Frambach RT, Schillewaert N: Organizational innovation adoption: a multi-level framework of determinants and opportunities for future research. Journal of Business Research. 2001, 55: 163-176. 10.1016/S0148-2963(00)00152-1.
56. Institute for Healthcare Improvement: The Breakthrough Series: IHI’s Collaborative Model for Achieving Breakthrough Improvement. IHI Innovation Series white paper. Boston: Institute for Healthcare Improvement; 2003.
57. Institute for Healthcare Improvement: Going Lean in Health Care. Going Lean in Health Care. IHI Innovation Series white paper. Cambridge, MA: Institute for Healthcare Improvement; 2005.
58. Grol R, Wensing M, Eccles M: Improving Patient Care: The Implementation of Change in Clinical Practice. 2005, Edinburgh, Scotland: Elsevier
59. Glisson C, Schoenwald SK: The ARC organizational and community intervention strategy for implementing evidence-based children's mental health treatments. Mental health services research. 2005, 7: 243-259. 10.1007/s11020-005-7456-1.
60. Estabrooks CA, Thompson DS, Lovely JJ, Hofmeyer A: A guide to knowledge translation theory. J Contin Educ Health Prof. 2006, 26: 25-36. 10.1002/chp.48.
61. Pronovost PJ, Berenholtz SM, Needham DM: Translating evidence into practice: a model for large scale knowledge translation. BMJ. 2008, 337: a1714-10.1136/bmj.a1714.
62. Cruess SR, Cruess RL, Steinert Y: Role modelling–making the most of a powerful teaching strategy. BMJ. 2008, 336: 718-721. 10.1136/bmj.39503.757847.BE.
63. Greenhalgh T, Robert G, Bate P, Kyriakidou O, Macfarlane F, Peacock R: How to Spread Good Ideas. Book How to Spread Good Ideas (Editor ed.^eds.). 2004, City: National Co-ordinating Centre for NHS Service Delivery and Organisation R & D.
64. Maidique MA: Entrepeneurs, champions and technological innovation. Sloan Manage Rev. 1980, 21: 59-76.
65. Schon DA: Champions for radical new inventions. Harv Bus Rev. 1963, 41: 77-86.
66. Pearson ML, Wu S, Schaefer J, Bonomi AE, Shortell SM, Mendel PJ, Marsteller JA, Louis TA, Rosen M, Keeler EB: Assessing the implementation of the chronic care model in quality improvement collaboratives. Health Serv Res. 2005, 40: 978-996. 10.1111/j.1475-6773.2005.00397.x.
